# Supplementary material for: Knowledge mobilisation of rapid evidence reviews to inform health and social care policy and practice in a public health emergency: Appraisal of the Wales COVID-19 Evidence Centre processes and impact, 2021–23
Source: PLoS One. 2024 Nov 26;19(11):e0314461. doi: 10.1371/journal.pone.0314461 (PMC11594520; doi:10.1371/journal.pone.0314461)
Supplement: Attachment — Submitted filename: Response to Editor and Reviewers_October 2024.docx [file pone.0314461.s002.docx]

Dr Micaela Gal

Health and Care Research Wales Evidence Centre

School of Medicine

Cardiff University,

Wales, United Kingdom

E-mail: [galm@cardiff.ac.uk](mailto:galm@cardiff.ac.uk). Tel +44 07455 307237

**Response to Editor and Reviewers**

22^nd^ October 2024

**Reference:** [PONE-D-23-22096] - [EMID:96a08200132b89a3]

**Knowledge mobilisation of rapid evidence reviews to inform health and social care policy and practice in a public health emergency: appraisal of the Wales COVID-19 Evidence Centre processes and impact, 2021-23**

Dear PLOS ONE Editor and Reviewers,

We wish to thank the editor who was involved in reviewing our manuscript, and both of the reviewers for their time and effort in reviewing our manuscript so carefully and for providing very helpful suggestions and comments to the authors.

We believe that addressing the suggestions has significantly strengthened the manuscript, and amplified its contribution to learning lessons and informing health and social care policy and practice during future infectious disease public health emergencies.

We have submitted a ‘marked up’ version of the original article (revised article with changes highlighted) and a clean version of the revised manuscript (manuscript), which address the editor and both reviewers’ comments. The explanations on how we have addressed both the initial editor and the 2 reviewer’s suggestions and comments are provided in the 3 tables below.

Thank you for your time and consideration of the revised manuscript.

Yours sincerely,

(Micaela Gal)

Table of Contents

[Table 1. Editor suggestions and author responses	2](#_Toc180574482)

[Table 2. Reviewer 1 comments and author responses	7](#_Toc180574483)

[Table 3. Reviewer 1 further comments and author responses	15](#_Toc180574484)

[Table 4. Reviewer 2 comments and author responses	19](#_Toc180574485)

[Appendix 1. Initial Letter to the Author from PLOS ONE	24](#_Toc180574486)

[Appendix 2. Second Letter to the Author from PLOS ONE	28](#_Toc180574487)

**Note**: The references to pages and line numbers given below, refer to the ‘marked up’ version of the manuscript (named: Revised article with changes highlighted).

# **Table 1. Editor suggestions and author responses**

| **No** | **Reviewer**  **or**  **Author** | **Comments and responses** |
| --- | --- | --- |
| **1** | Editor suggestion | **Enhancement of Methods Section:** Reviewers suggested providing more detail on stakeholder engagement, collaborative work processes, and data collection methods to offer readers a clearer understanding of the WCEC's approach. |
|  | Author response | We agree that more detail is needed in the methods section related to stakeholder engagement, collaborative work processes and data collection.  We have included more information on each of the suggestions for improving the methods, and provide evidence of this in our responses to Reviewer 1 (in Table 2, (below)).  We have also added 4 additional references (References 18-21) from the Evidence Centre, which cover in-depth the overall approach used by the Centre for stakeholder identification and engagement, identification and prioritisation of the research questions, and how the stakeholders and evidence centre team worked together collaboratively during the evidence review process. |
| **2** | Editor suggestion | **Improvement in Presentation:** Incorporating more visuals, simplifying language, and exploring alternative presentation methods such as videos or poems could make the information more accessible and engaging for a wider audience. |
|  | Author  response | **Incorporating more visuals:**  We have included 2 additional tables and 2 figures to enhance and explain the text. These are in line with the comments from the reviewers:  **Table 2. Information contained within the front pages of the Centre reports** (page 11)  **Table 5. Examples of associated activities conducted by the WCEC** (page 22)  **Fig 2. Questions received, prioritised, and accepted onto the Centre work programme** (page 9)  **Fig 4. Evidence Centre rapid review (evidence synthesis) outputs** (page 20)  **Simplifying language:**  We have reviewed the manuscript and simplified language used to make it more accessible.  **Alternative presentation methods:**  The suggestions to use alternative methods of presentation such as videos or poems relate to Feedback provided by Reviewer 2. This was helpful and insightful. Indeed, one of our public group members had suggested poetry for one of our Evidence Centre reports, which focused on children and education during the pandemic. We have now included this in the discussion (page 32 line 610):  *A suggestion for wider dissemination of findings in a different format was made by a public member (e.g., review findings relevant to children and young people could be made into a poem). We acknowledge that wider dissemination methods such as creating stories, poems or videos would likely be valuable to make findings more accessible and engaging to wider groups. However, this was not possible at the time due to resource constraints (available staff time and additional funding).*  We have also included an additional reference (*40), which refers readers to a public facing legacy report for the Wales COVID-19 Evidence Centre (***Wales COVID‐19 Evidence Centre Legacy Report. Wales COVID-19 Evidence Centre. June 2023.** <https://researchwalesevidencecentre.co.uk/sites/default/files/2023-11/Wales_COVID-19_Evidence_Centre_Legacy_Report_June23_ENGLISH.pdf>)  We have included the following in our Discussion section (page 31, line 605):  *To make the work of the WCEC more widely accessible, the Centre also produced a publicly available legacy report.(40) This report includes information about the Centre structure, methods, reviews, knowledge mobilisation and impact. The lay summaries accompanying each review were written by members of the Centre public partnership group, and together with the infographics, increase accessibility of the review findings.* |
| **3** | Editor suggestion | Streamlining of Content: (1) Streamlining research questions and (2) aligning rapid review processes with publication criteria were recommended to ensure clarity and manage expectations effectively. |
|  | Author  response | **1. Streamlining research questions:**  The research questions, which the Centre received from the stakeholders were typically very broad. The Centre had to work with the stakeholders to manage their expectations, and streamline the questions to ensure that the reviews could deliver useful information and be completed within the stakeholder’s timeframe. The streamlining/focusing of the questions was part of the rapid evidence review process, and not part of the knowledge mobilisation processes of the Centre, which were the focus of this manuscript (and so was not described).  However, we agree that this point would be of interest to readers of this manuscript. Therefore, we have made reference to this in the Discussion section (Page 29, Line 542), and included a reference to another Centre manuscript, where the streamlining of research questions for the Evidence reviews is described in detail (below):  *The responsiveness and involvement of stakeholders and members of the public were key to focusing the research questions, which were usually too broad to be answered in the available amount of time.(23)*  **A bespoke rapid evidence review process engaging stakeholders for supporting evolving and time-sensitive policy and clinical decision-making: reflection and lessons learned from the Wales Covid-19 Evidence Centre 2021-23. Lewis R et al.** <https://assets-eu.researchsquare.com/files/rs-3878814/v1/59d98322-ac9e-492b-aead-afbd02db0b37.pdf?c=1706010939>  **2. Aligning rapid review processes with publication criteria:**  We acknowledge that this is an interesting point and important point.  Our rapid review processes including the methodology and report content are aligned to approved methods for conducting rapid reviews. The findings from a number of the rapid reviews have now been published in peer reviewed journals and met the publication criteria needed for these.  We have now made reference to this in the discussion (Page 36, Table 8, last box):  *When producing rapid reviews, the requirements and criteria of peer reviewed journals and the research and impact requirements of academia should be considered. Our review methodology is aligned to recognised rapid review methodology and a number of the reviews have now been published in peer reviewed journals.(23)*  The method for our rapid review process is also described in a referenced manuscript:  **A bespoke rapid evidence review process engaging stakeholders for supporting evolving and time-sensitive policy and clinical decision-making: reflection and lessons learned from the Wales Covid-19 Evidence Centre 2021-23. Lewis R et al.** <https://assets-eu.researchsquare.com/files/rs-3878814/v1/59d98322-ac9e-492b-aead-afbd02db0b37.pdf?c=1706010939> |
| 4 | Editor suggestion | **Stakeholder Engagement:** Continued close collaboration with stakeholders, including policy and practice decision-makers, was emphasized to enhance the utilization and impact of evidence reviews. |
|  | Author  response | Thank you for this helpful suggestion.  We had already included some information regarding this in the Discussion (page 28). We have included an additional paragraph this in the manuscript Discussion (page 29 line 556).  Fo*llowing the end of funding of the Wales COVID-19 Evidence Centre, we received 5 further years of funding, and are now funded as the ‘Health and Care Research Wales Evidence Centre (2023-2028). This enables us to continue close collaborations with the stakeholders we have worked with in the COVID-19 Evidence Centre to enhance use of our report findings, and collect and evidence longer term evidence of impact.* |
| 5 | Editor suggestion | Additionally, I would like to propose the inclusion of an article featuring an interview with Prof. Marianna Arvanitakis, the winner of the European Award for the Best Medical Practice in the COVID-19 Pandemic. This article could highlight how recognition of exemplary practices can serve as a valuable resource in crisis situations and inform future health and social care policies. available here: https://globalbioethicsenquiry.com/wp-content/uploads/2023/07/VP2-JASNA.pdf |
|  | Author  response | We agree that highlighting how examples of exemplary practice during the pandemic should serve as valuable resources and points of learning for future crisis situations, as well as informing health and social care policies going forward. We have made reference to this and added the citation (Page 3, Lines 78-81)  *There are some valuable examples of exemplary practices during the pandemic. These should serve as a valuable resource and lessons for managing future crisis situations, and to inform future health and social care policies.*(3) |
| 6 | Editor suggestion | Please ensure that your manuscript meets PLOS ONE's style requirements, including those for file naming. |
|  | Author  response | We have ensured that the revised manuscript meets PLOS ONE style requirements, including for the naming of the files.  We have also used the Preflight Analysis and Conversion Engine (PACE) digital diagnostic tool, to ensure that figures meet PLOS ONE style requirements. |
| 7 | Editor suggestion | Data sharing for the study. 2. We note that you have indicated that there are restrictions to data sharing for this study. For studies involving human research participant data or other sensitive data, we encourage authors to share de-identified or anonymized data. However, when data cannot be publicly shared for ethical reasons, we allow authors to make their data sets available upon request. Before we proceed with your manuscript, please address the following prompts:  a) If there are ethical or legal restrictions on sharing a de-identified data set, please explain them in detail (e.g., data contain potentially identifying or sensitive patient information, data are owned by a third-party organization, etc.) and who has imposed them (e.g., a Research Ethics Committee or Institutional Review Board, etc.). Please also provide contact information for a data access committee, ethics committee, or other institutional body to which data requests may be sent.  b) If there are no restrictions, please upload the minimal anonymized data set necessary to replicate your study findings to a stable, public repository and provide us with the relevant URLs, DOIs, or accession numbers. … You also have the option of uploading the data as Supporting Information files, but we would recommend depositing data directly to a data repository if possible.  Please update your Data Availability statement in the submission form accordingly. |
|  | Author Response | We agree that research data should be made available wherever possible.  The anonymised survey data (questions and responses (answer selection and free text) from the Stakeholder survey has now been made avaialble on the repository Figshare (<https://figshare.com>). The data file can be downloaded here:  <https://figshare.com/articles/dataset/Wales_COVID-19_Evidence_Centre_2021-2023_Stakeholder_survey/25912459>  Some data and identifiers were removed before adding the data to Figshare (i.e. names. job title, place of work, comments that may identify participants, feedback where participants did not give permission to quote this). This was done to ensure the data was anonymised, as respondents had not consented to have this data shared without anonymisation.  We have included this information in the Results section – 3.5.1 Stakeholder survey (Page 24, Line 491):  *The data from the* [*Stakeholder survey*](https://figshare.com/articles/dataset/Wales_COVID-19_Evidence_Centre_2021-2023_Stakeholder_survey/25912459) *are accessible on the data repository Figshare (Wales COVID-19 Evidence Centre 2021-2023 Stakeholder survey).*  The Data Availability statement in the submission form has been updated accordingly. |

Our responses to the reviewers are provided in the tables 2,3 (Reviewer 1) and 4 (Reviewer 2) below.

# **Table 2. Reviewer 1 comments and author responses**

| **No** | **Reviewer**  **or**  **Author** | **Comments and responses** |
| --- | --- | --- |
| **8** | **Reviewer comment** | Thank you for the opportunity to review this manuscript, and congratulations to the WCEC for the important work they completed during the COVID-19 pandemic. I appreciate how difficult these types of reports are to write, as they don't fit into the traditional IMRaD format of our typical scientific papers.  Overall, I would like to have more detail within the methods section to better understand what steps the WCEC undertook as part of their work and KM. I have provided some specific examples of this below. |
|  | **Author response** | - We would like to thank the reviewer for the detailed review of our manuscript, and for the very helpful suggestions on how we can improve this, especially the methods section. - Below, we describe how we have addressed each point raised. |
| **9** | **Reviewer comment** | **Background** - Page 4, Lines 100-102 - The authors make an important point that research evidence is not the only factor that decisions should be made on. I think it would be important to note that values/preferences (of individuals and communities) and also resources (financial, human, etc.) also play an important role in decision-making. |
|  | **Author response** | We agree that these are important factors that are additionally included in decision-making. We have added the following (now at Page 5, Line 116-117):  *In addition, the values and preferences of individuals and communities, and available human and financial resources also play an important part in decision making.* |
| **10** | **Reviewer comment** | - Page 4, Line 103 - this sentence is a bit awkward (achieving impact is enabled...) |
|  | **Author response** | We agree and have revised this sentence, which now reads as follows (Page 5, Line 118):  *Achieving impact from research is supported by knowledge mobilisation processes, and plans on how to achieve accessibility and dissemination of research findings should underpin the research from the start.* |
| **11** | **Reviewer comment** | Figure 1: I like the use of the KTA framework, but it would be helpful to have more of the original steps maintained in the figure so the reader can see how the WCEC applied/adapted it for their KM purposes. |
|  | **Author response** | Thank you, and we agree with this helpful suggestion.  We have revised the figure, and made alterations to align our figure better with the steps of the original Knowledge to Action (KTA) framework. This makes it clearer for readers to see how we have applied and adapted the KTA framework for our knowledge mobilisation purposes.  The changes we have made include changing some of our wording to that used in the original framework where appropriate, for example:   - Added a box with the following information ‘*Identify problem – Identify, review, select knowledge*’ - ‘Evidence Creation’ has been changed to ‘*Knowledge* Creation’ - ‘Evidence Outputs’ has been changed to ‘*Knowledge products’* - ‘Evidence need’ has been changed to *‘Knowledge inquiry (Evidence need)’* - Step 5 Track and evidence impact. We have added to this box: ‘Track and evidence impact *(Monitor knowledge use and evaluate outcomes)*’ - ‘Identify and address barriers to knowledge mobilisation’. We have changed this to ‘*Assess and address barriers to knowledge use* and mobilisation’ - ‘Outputs: Reports with topline summary, infographics, lay summaries, presentations, newsletters, blogs. We have changed this to ‘*Adapt knowledge to local context: Targeted* outputs: *Reports with topline summary (including local policy and practice implications), infographics, lay summaries, presentations)’.* |
|  |  | **METHODS SECTION** |
| **12** | **Reviewer comment** | **1.** It would be helpful for the authors to provide more detail on how the various stakeholder groups (Table 1) were engaged. How were questions identified and prioritized? |
|  | **Author response** | The initial identification and engagement with stakeholders to identify and prioritise the questions, which the Centre would answer, was not part of the knowledge mobilisation processes described in this manuscript.  However, we very much appreciate that these points were picked up by the reviewer as it highlights where our methods could be explained more clearly and where we need to point readers to literature where the WCEC methods are described in detail.  To this end, we have added 4 further references produced by the Evidence Centre team, which cover in-depth the methods related to stakeholder identification and engagement, identification and prioritisation of research questions, and how the stakeholders and evidence centre team worked together collaboratively during the review process: (Methods Section 2.1 (page 7, references for lines 164-166)):   - **In-depth description of the initial stakeholder engagement process:**   Informing evidence-based policy during the COVID-19 pandemic and recovery period: learning from a national evidence centre. Glob Health Res Policy. 2024 May 31;9(1):18. doi: 10.1186/s41256-024-00354-1. PMID: 38822437; PMCID: PMC11140997. <https://pubmed.ncbi.nlm.nih.gov/38822437/>   - **Identifying and prioritising questions for the Evidence Centre Work Programme:**   Working with stakeholders to rapidly identify and prioritise COVID‐19 health and social care evidence needs for evidence‐informed policy and practice decision making: processes, results, and lessons from the Wales COVID‐19 Evidence Centre \| Research Square BMC Health Research Policy and Systems (under review). <https://www.researchsquare.com/article/rs-3286253/v1>   - **Working with members of the public to identify priority questions for the WCEC Work Programme:**   Involving the public in research prioritisation and as research partners in the Wales COVID‐19 Evidence Centre (2021–23): processes, research priorities, outputs, and lessons \| Research Square Research Involvement and Engagement (under review). <https://www.researchsquare.com/article/rs-3624819/v1>   - **Conducting the evidence reviews (rapid evidence summaries, reviews and evidence maps), and how stakeholders were engaged and involved in this process:**   A bespoke rapid evidence review process engaging stakeholders for sup‐ porting evolving and time‐sensitive policy and clinical decision‐making: reflection and lessons learned from the Wales Covid‐19 Evidence Centre 2021–23 \| Research Square BMC Health Research Policy (under review). <https://www.researchsquare.com/article/rs-3878814/v1> |
| **13** | **Reviewer comment** | **2.** I would like to read more about how the WCEC and stakeholders worked collaboratively (page 7, Lines 155-162). Were three online meetings completed per review or over the course of the WCEC operations? |
|  | **Author response** | Thank you for highlighting the need to further explain this for readers.  We have added a paragraph (on Page 8/9 Lines 186 to 195), and an additional reference (below), which details the review process and how the WCEC worked collaboratively with the stakeholders as part of the process:  *During the review process, 1-3 representative stakeholders from each stakeholder group attended a minimum of three on-line meetings with the WCEC including the collaborating partner review team. These meetings were part of every review conducted by the Centre. The meetings included an initial meeting to ensure the question was focused and that the outcomes were relevant to the stakeholders needs, a second meeting (one week later) to present initial findings and discuss the next review steps, and a third meeting to present findings and discuss knowledge mobilisation and impact.(22) If a key stakeholder was unable to attend a meeting, they were invited to send a representative from their group to attend on their behalf.*  **Reference added:**  A bespoke rapid evidence review process engaging stakeholders for sup‐ porting evolving and time‐sensitive policy and clinical decision‐making: reflection and lessons learned from the Wales Covid‐19 Evidence Centre 2021–23 \| Research Square BMC Health Research Policy (under review). <https://www.researchsquare.com/article/rs-3878814/v1> |
| **14** | **Reviewer comment** | **3.** What are some concrete examples of capacity building, sharing methodologies, and identifying gaps as part of the "associated activities" - maybe a table listing these would be helpful? |
|  | **Author response** | Thank you for highlighting the need for further information.  We have included concrete examples of ‘Associated activities’ in the results section ‘Associated Activities 3.4’ (page 21).  We have adjusted the wording in this section and added an additional Table (Table 5, page 22) to this section to help clarify this. |
| **15** | **Reviewer comment** | **4.** Who were the stakeholders who were invited to take part in the survey? Were there any eligibility criteria set out? How were they invited to participate? |
|  | **Author response** | We have added some further information to clarify this (Section 2.5.1 Stakeholder survey, page 15, lines 337):  *All stakeholders (n=44) taking part in the three stakeholder meetings during the review process were asked to complete the survey. Stakeholders were asked to completed the survey via e-mail following the report publication, and up to three e-mail reminders were sent.* |
| **16** | **Reviewer comment** | **5.** What is meant by "accepted onto the work program" (page 13, line 282-4? Only these questions were taken on by the WCEC or only these questions were evaluated through the survey? |
|  | **Author response** | We agree that this information needed clarification.  We have added some additional information to clarify this (Page 16, Line 346):  *The WCEC received a total of 223 questions from stakeholders. These included 11 urgent Welsh Government questions. Only questions (n=58) with a clear pathway-to-impact (e.g., answering a question would help to inform Welsh Government advisors, a policy or plan) were accepted onto the WCEC work*  *programme (research to be conducted by the Centre. (19)*  We have also added an additional Figure), and a reference to a relevant WCEC publication explaining the question prioritisation process and work programme development (below):   - ***Fig 2. Questions received, prioritised and accepted onto the Centre Work Programme.*** - ***Reference:*** Working with stakeholders to rapidly identify and prioritise COVID‐19 health and social care evidence needs for evidence‐informed policy and practice decision making: processes, results, and lessons from the Wales COVID‐19 Evidence Centre \| Research Square BMC Health Research Policy and Systems (under review). <https://www.researchsquare.com/article/rs-3286253/v1> |
| **17** | **Reviewer comment** | **6.** More detail could be added as to how citations were identified, how email and meeting sessions were used to capture data when surveys were incomplete. |
|  |  | We agree that this information would be of interest to the readers.  **How citations were identified:**  We have added some more detail to section (2.5.2 Feedback and collection of metrics) about how citations of our reviews were identified in Welsh Government publications (page 16, line 359):  *Identifying reference to WCEC reports in Welsh Government publications was enabled by having a boundary-spanning Welsh Government team member in the core group to help identify and search for these. Members of the WCEC core team also searched reports published on the TAC/TAG pages of the Welsh Government website for inclusion of WCEC reports.*  We have also added some more information to same section (2.5.2 Feedback and collection of metrics) about how e-mail and meeting sessions were used to capture data when surveys were not completed (page 16 line 351):  *Where stakeholder surveys were not completed, information pertaining to impact and use of the report was collected via e-mail requests to the stakeholders involved in the stakeholder meetings, and during discussions at the on-line stakeholder meetings and the evidence briefing sessions, which were both attended by stakeholders. Where further information was needed and stakeholders did not respond to e-mails request or complete the survey, the boundary-spanning WG team members contacted WG stakeholders directly to obtain this information.* |
|  |  | **RESULTS SECTION** |
| **18** | **Reviewer comment** | 1. What is meant by "good engagement" - how was this determined vs. poor engagement for example? (page 13, line 294). 2. Did all stakeholders take part in all steps (lines 295-299) |
|  | **Author response** | **1 and 2.** We have clarified this by adding information to section 3.1 (page 117. Lines 373), which now reads:  *We defined good engagement as stakeholders or stakeholder representatives i) attending stakeholder meetings to help refine the research question (usually ‘PICO’ format(24)) and agreeing relevant outcomes, ii) agreeing deadlines for the evidence outputs, iii) interpreting review findings, identifying policy and practice implications, iv) providing input to knowledge mobilisation plans, v) supporting dissemination (e.g., sharing reports with colleagues) and vi) providing information on how the research evidence was used. Where requested, stakeholders or stakeholder representatives also participated in the additional dissemination activities (evidence briefings and public symposia). Poor engagement would have been defined by failure to attend (personally or via sending a representatives) the stakeholder meetings during the review process without which the review would not have been taken forward, and providing no contribution towards knowledge mobilisation or evidence of how the review findings were used.* |
| **19** | **Reviewer comment** | 3. How many reviews were undertaken in total? How many were evaluated through the survey and how many through the other supplementary data collection methods? |
|  | **Author response** | We have added the following lines and a reference to provide information to answer these questions:  **How many reviews were undertaken in total:**  *In two years of operation (March 2021–23), the WCEC engaged with 44 stakeholder groups and produced 51 reports from its rapid review work.(19)* (Section 3.1, line 369)  **How many reviews were evaluated via the survey and how many through other supplementary data collection methods:**  *22 of 51 reviews were evaluated via the survey, and the remainder were evaluated through data collection following e-mail request, feedback at stakeholder meetings, and feedback provided by stakeholders to the WG liaison member of the core team.* (Section 3.5.1 Stakeholder survey line 466 ) |
| **20** | **Reviewer comment** | 4. Can you provide demographic data for survey respondents? |
|  | **Author response** | The 21 survey respondents included policy makers, government scientific advisors, clinicians, Social Care Wales leads (our stakeholders) who had submitted a question to the Centre and had participated in the review process, including attending the stakeholder meetings. This was the only criteria for survey participation.  It might have been interesting to collect demographic data from them, but we did not do this. |
| **21** | **Reviewer comment** | 5. I believe the quotations come from the open ended questions in the survey. How was this qualitative data analysed? (it is not mentioned in the methods) |
|  | **Author response** | Thank you for highlighting this, and we agree that we should have provided information about how the survey results were analysed in the methods section (2.5.1 Stakeholder Survey, page 15, line 332)  *Participants were asked to select from a 5-point Likert scale for questions, and the data was analysed to calculate the frequency percentage. The survey also included open-ended questions to collect stakeholder feedback. The open-ended questions were optional and information was used to gather information for knowledge mobilisation plans and as example quotations (anonymised).*  We have also provided the following information at the beginning of the Results section (Line 366):  *Note that quotes presented in the results section are taken from the stakeholder survey, and are for illustrative purposes only.* |
| **22** | **Reviewer comment** | 6. Similar comment on Page 14, Lines 320, what is meant by "worked well" - how was this determined? Two examples are given, but it may be helpful to have this information collated across all reviews and a summary presented |
|  | **Author response** | **(3.2.1 Knowledge mobilisation plans.** Page 18, lines 405)  The following information was added to the two examples of ‘what worked well’ already given:  Gathering information to support a knowledge mobilisation plan for each review worked well during discussions at stakeholder meetings, evidence briefings and via e-mail follow-up. *For all the reviews, stakeholders or their representatives provided suggestions about which organisations and persons would be interested in the findings, and shared the reports with their groups.*  **Collating information across all reviews**:  The WCEC knowledge mobilisation plan actions and processes were iterative and flexible to meet the needs of the different stakeholders. Therefore, not all processes e.g. evidence briefings, presentations to Welsh Government and other stakeholders wider groups, public symposia and preparation of infographics were requested by stakeholders and carried out for each review.  Therefore, collating representative information across all reports would not work for all the knowledge mobilisation processes. For example:   - Only 24 evidence briefing sessions were held although a total of 51 reviews were conducted, as the WCEC was only asked to do the Welsh Government evidence briefing sessions half-way through its lifetime. - The WCEC public symposia and social media blogs were themed, and so not all review findings were shared via this method. - Not all stakeholders asked for the reports to be presented to their wider teams. - Not all stakeholders requested infographics. - Early reports were published only in the WCEC library rather than on preprint servers.   **Table 3. ‘Results from stakeholder informed knowledge mobilisation activities’** provides information on revies conducted and reports, infographics and presentation of review findings including figures. |
| **23** | **Reviewer comment** | Table 2 1. I now see that information on the 51 completed reviews is listed in Table 2 but I think that information should be a key feature in the results 2. Timeline of completion - is it possible to give a mean or IQR rather than "usually 1 week to 3 months" 3. The authors refer to different parts of the report, (topline summary, etc.). It would be helpful to have described earlier what a typical report included. 4. This table lists some examples of impact, but it would be more comprehensive if this was collated across reviews, rather than just choosing a few examples (which may be perceived as biasing the anticipated impact) |
|  | **Author response** | **1.** Table 2 (now **Table 3, Results from the stakeholder informed knowledge mobilisation activities’**) is the first Table to appear in the Results section. It is in this position as the Results and Methods sections follow the order of the steps in our knowledge mobilisation framework (i.e. number 1 -stakeholder engagement, number 2 - knowledge mobilisation plans, and number 3 - outputs and presentations (Table 3). We believe  **2**. Relating to times of review completion, we have added the following information (Table 3, page 19, top box):  From April 2021 to March 2023, the WCEC conducted 51 evidence reviews and published 51 reports (in the WCEC library and on pre-print servers), which met stakeholders timelines. *(The 51 evidence reviews included 10 rapid evidence summaries and 6 rapid evidence maps (delivered within 2 months), and 35 rapid reviews (delivered within 3 months). Where very urgent, e.g. a review about the safety of ozone machines in schools, an evidence summary was delivered in 1 week).*  We did not record the exact times (number of days) it took for each review. Some reviews were also expedited and prioritised for editing and publishing in the WCEC report library if the information was required more urgently by the stakeholders.  **3.** We agree that it would be useful to have information about what a typical report included at an earlier point in the manuscript. We have added the information below (Section 2.2.2 Reports, line 237), and included a Table (Page 11) showing what information was provided in the front 3 pages (Review details and Topline summary) of all our reports:  *Reports were full scientific evidence review reports, but to aid access to key information by stakeholders, each report had 3 specific front pages. These included a short information box of review details, followed by a 2-page ‘Topline summary’ containing key information in an accessible language (Table 2). Stakeholders who attended the meetings during the review process, contributed and reviewed the Topline summaries to ensure these contained the key information they needed, that the policy and practice implications were accurately reflected, and that the findings were accessible.*  **4.** We believe that the Reviewer is referring to what is now Table 7, ‘**Examples of WCEC rapid reviews: impact and pathway to impact’** (Section 3.5.2. page 25).  In this Table we included 15 examples of 21 reviews, which were ‘referenced in Welsh Government advice and reports’. We have added the following information:  *Twenty-one review reports were referenced in Welsh Government advice and reports, and informed policy and practice decisions.* *The best impact examples of review findings being used to inform policy and practice decisions, are provided in Table 7.*  In other cases, it was challenging to evidence impact, i.e. reviews which identified a lack of published evidence, or the evidence was of low quality. We have described this, and also added a line (line 512):  *Evidencing actual impact from these types of reviews was challenging and often not possible.*  We hope that this addresses the reviewer’s recommendations, and believe that a lengthy Table for all 51 reviews would not add any further significant information. |
| **24** | **Reviewer comment** | I'm not clear the distinction between survey results and the "feedback and collection of metrics" section 3.5.2 - how was this data collected if not through the survey? |
|  | **Author response** | The sections ‘3.5.1 Stakeholder survey’ and ‘3.5.2 Feedback and collection of metrics’ are both included under ‘3.5 Track and Evidence impact.  In section ‘3.5.2 Feedback and collection of metrics’, we included feedback from stakeholders, who did not complete the survey (i.e. feedback was collected via e-mail, at stakeholder meetings and the WG evidence briefing sessions).  We agree that the way these headings are described can lead to confusion. We have now changed the heading of 3.5.2 to read:  **3.5.2 Collecting additional stakeholder feedback and metrics** |
| **25** | **Reviewer comment** | Table 6 - how was this list compiled? Was a certain process used, or would this be better placed as part of the discussion? In fact, much of it is repeated in the discussion. |
|  | **Author response** | Table 6 (now Table 8), is **‘Learning from the WCEC knowledge mobilisation and impact processes’.**  On reflection, we agree with the reviewer that this table and the section related to it are best placed within the discussion.  No specific process was used to compile this list, but we gathered and noted challenges and solutions (enablers) throughout the lifetime of the Centre.  We have moved the Table into the Discussion section and merged this with the previous table under **‘Lessons learn by the Evidence Centre’** (Table 8 page 33). |
|  |  | **OTHER COMMENTS** |
| **26** | **Reviewer comment** | Minor suggestions for the authors' consideration **1.** There are a number of longer sentences, particularly in the background which could be broken up to enhance readability.  **2.** Page 3, Line 88, spell out the acronym for BRIDGE criteria |
|  | **Author response** | We thank the reviewer for picking up on these points.  **1.** We have reviewed and revised the manuscript to enhance readability and this included breaking up lengthy sentences.  2**.** As BRIDGE is not an actual acronym but comes from the ‘Bridge Building Model’, we have changed the sentence (Line 96) to read as follows and inserted a reference to refer readers to this:  *‘..These including the widely used and adapted ‘Knowledge to Action’ Framework, Developing Evidence Enriched Practice, the ‘What Works Networks’, and the ‘Bridge Building Model’.’(5)* |

# **Table 3. Reviewer 1 further comments and author responses**

| **No** | **Reviewer**  **or**  **Author** | **Comments and responses** |
| --- | --- | --- |
| **1** | **Reviewer comment** | **Was ethical approval sought or received for this survey? It is not mentioned anywhere** |
|  | **Author response** | The stakeholder survey was an evaluation of the services provided by the Evidence Centre. It requested feedback from those who had used the services in order to evaluate and optimise our processes and output, and evaluate impact.  This was clearly a service evaluation and not a research survey, hence no ethical approval was required.  To explain this in the manuscript, we have added a sentence (Section 2.5.1 Stakeholder survey)  *‘No ethical approval was required for this survey as it was a service evaluation of the WCEC processes and outputs, and not conducted for research purposes’.* |
| **2** | **Reviewer comment** | **There are some inconsistencies in the use of acronyms throughout (e.g., TAC on line 160 and spelled out on line 165).** |
|  | **Author response** | We have checked through the manuscript and refer to Technical Advisory Cell (TAC) in the first instance that this is mentioned in the manuscript, followed by the ‘TAC’ abbreviation subsequently. |
| **3** | **Reviewer comment** | **Line 172 - were questions received and prioritized at one time or on a rolling basis? I am guessing the later but it is not clear.** |
|  | **Author response** | We have clarified this as follows (from line 182 in marked up manuscript copy):  *‘The WCEC contacted stakeholders to invite new questions every 3 months. Additionally, the prioritisation process was flexible and responsive to accommodating new urgent questions outside the 3-monthly invite.’* |
| **4** | **Reviewer comment** | **Line 182 - what is the collaborating partner review team?** |
|  | **Author response** | We agree that this was confusing. The collaborating partner review team were members of the WCEC. As they are included in references to the WCEC in other parts of the manuscript, we have removed the words’ collaborating partner review teams.  The sentence (line 189) now reads:  ***‘****During the review process, 1-3 representative stakeholders from each stakeholder group (that had a question accepted onto the WCEC work programme) were invited and attended a minimum of three on-line meetings with the WCEC.’* |
| **5** | **Reviewer comment** | **Methods - I'm not sure if collaborating and co-production are the appropriate terms based on what is described (which is understandable given the very tight timelines the team was working on). For example, using the IAP2 framework for engagement, what the authors describe sounds more like "consult" or maybe "involve". Perhaps a definition of co-production and collaboration with citation would be helpful.** |
|  | **Author response** | We agree that collaboration and co-production are not what would be as defined by the International Association for Public Participation (IAP2) framework  (https://www.iap2.org/page/pillars).  We believe that collaboration and coproduction are appropriate terms to describe our novel methods of working with and involving our stakeholders (key health and social care policy and practice decision makers). We describe how they are involved and work with us in the manuscript, and have also included the following clarification (page 6, line 142 - 150):  *In this paper we have defined collaboration as an interdisciplinary cross-sector partnership that involves working together and coordinating between researchers, and stakeholders from institutions and or organisations, (e.g. WCEC and Welsh government), which brings distinct and essential expertise to a project. We have defined co-production as working together with our stakeholders throughout a project (e.g. a rapid review), in processes including attending meetings, refining the research questions, identifying outcomes, discussion and interpretation of the findings, interpreting policy and practice implications, reviewing and commentating on all outputs, (e.g. reports, infographics), and* *involvement in knowledge mobilisation processes.* |
| **6** | **Reviewer comment** | **Section 2.4 - the authors mention gaps were "collated" - how was this done, and what was done with this information? are there any examples you can provide?** |
|  | **Author response** | We have provided a clearer explanation in the manuscript, in section 2.4:  *‘Evidence gaps and need for further research identified during the review processes were ~~collated~~ clearly indicated within each report, and collected in a document, with an aim to share with research funders (e.g., Health and Care Research Wales and the National Institute of Health Research).*  As these evidence gaps were not shared with funders during the lifetime of the WCEC we cannot include an example.  (Research gaps have been subsequently suggested to the National Institute of Health Research, (via Health and Care Research Wales) to consider for research funding areas). |
| **7** | **Reviewer comment** | **Line 348 seems to be missing something ("agreeing relevant outcomes")** |
|  | **Author response** | **This sentence is now at line 372 and we have made a change to clarify this (underlined):**  Good engagement was defined as stakeholders or stakeholder representatives i) attending stakeholder meetings to help refine the research question (usually ‘PICO’ format(24)) and agreeing relevant ~~objectives~~ research outcomes, ii) agreeing deadlines for the evidence outputs, iii) interpreting review findings, identifying policy and practice implications, iv) providing input to knowledge mobilisation plans, v) supporting dissemination (e.g., sharing reports with colleagues) and vi) providing information on how the research evidence was used. |
| **8** | **Reviewer comment** | **The authors define good vs. poor engagement, but this probably fits better in the methods (if it was prespecified) vs. the results** |
|  | **Author response** | The survey was conducted as a service evaluation and not research. A method to evaluate good vs poor engagement with the survey was not prespecified. As such we feel that this fits better as the comment in the results section. |
| **9** | **Reviewer comment** | **Line 359 - n = 21 responded to the survey, how many were invited?** |
|  | **Author response** | **44 stakeholders were invited, and 21 responded to the survey (48%).**  **We have added this information in section 2.5.1:**  ‘*All stakeholders (n=44) taking part in the three stakeholder meetings during the review process were asked to complete the survey’.*  **Also, within the results section 3.5.1:**  *All stakeholders (n=44) who participated in the reviews and stakeholder meetings (1-3 per evidence review) were invited to complete the survey. However, stakeholders were variably and overall less engaged in the survey processes. Twenty-one (48%) stakeholders (mainly from Welsh Government) completed the survey (to December 2022) and two of these completed the survey for more than one evidence review.* |
| **10** | **Reviewer comment** | **The authors have provided more information as to how the qualitative data were collected, but there is no description of data analysis approaches for the qualitative (or quantitative data for that matter)** |
|  | **Author response** | **The survey was conducted as a service evaluation and not research. We had already added information about how the quantitative survey data was analysed. We have added additional information about the qualitative information in section 2.5.1:**  *‘Participants were asked to select from a 5-point Likert scale for questions, and the data was analysed to calculate the frequency percentage. The survey also included open-ended questions to collect stakeholder feedback. The open-ended questions were optional and information was used to gather information for knowledge mobilisation plans and as example quotations (anonymised). No qualitative analysis was planned or conducted’.* |
| **11** | **Reviewer comment** | **Line 439 - how many of the 21 surveys were incomplete?** |
|  | **Author response** | **2 of the 21 surveys were incomplete. We have added this information to section 3.5.1:**  *‘Two ~~Some~~ respondents dropped off towards the end of the survey.’* |
| **12** | **Reviewer comment** | **Table 7 could be integrated with Table 4, perhaps as a row of "policy impacts"; there is already some duplication in the table (e.g., infographics for midwives).** |
|  | **Author response** | We do not think these tables should be integrated.  The tables are used to illustrate distinct steps of the WCEC processes. Table 4 describes the results of wider dissemination activities, while table 7 describes impact.  Regarding duplication of the WCEC outputs which was used by midwives, Table 4 focuses on the infographic produced as part of the outputs for the review, whereas Table 7 describes the overall impact of the review. |
| **13** | **Reviewer comment** | **. With respect to facilitators to the impact or how to better mobilize next time, where did the resources come from to develop this centre?** |
|  | **Author response** | **In section 1.1 we describe the Centre and also include information on the funding:**  *‘With the remit of ‘Good questions, answered quickly’, the WCEC was funded by Welsh Government and rapidly set-up (March 2021 – March 2023’).* |
| **14** | **Reviewer comment** | **Table 8 - i'm unclear if these are part of the "results" (under the objective to provide recommendations for best practice" or discussion. Perhaps if the authors were more clear on whether these are personal reflections or more informed by data.** |
|  | **Author response** | We have renamed Table 8 as ‘**Reflecting on the learning from the WCEC knowledge mobilisation and impact processes**.’  The second column is labelled as ‘**Enablers, challenges and reflection’**  **We have added ‘***Our reflections are provided in Table 8.’* |
| **15** | **Reviewer comment** | **The strengths/limitations section should focus more on the strengths/limitations of this paper and its evaluation rather than the centre as a whole.** |
|  | **Author response** | Thank you for this observation and suggestion. We have moved a paragraph describing some lessons learnt from the strengths and limitations section, to the section of ‘Lessons learnt’ in the discussion. |
| **16** | **Reviewer comment** | **Another pass for readability is advised... for example "it is worth noting" is on line 544 and line 551.** |
|  | **Author response** | We have gone through the manuscript again to help ensure readability. |

# **Table 4. Reviewer 2 comments and author responses**

| **No** | **Reviewer**  **or**  **Author** | **Comments and responses** |
| --- | --- | --- |
| **27** | **Reviewer comment** | Reviewer #2: This scoping review focuses on the role of care managers and the knowledge mobilization steps undertaken by the Welsh Centre for Evidence and Dissemination (WCEC) during the COVID-19 pandemic. The WCEC engaged closely with stakeholders, utilized the Knowledge to Action framework, and successfully disseminated evidence reviews to inform health and social care decision-making. However, some significant concerns need to be addressed before publication. |
|  | **Author response** | - We would like to thank the reviewer for the careful and detailed review of our manuscript, and for the very helpful suggestions on how we can improve the manuscript, especially the methods section. - Below, we describe how we have addressed each point raised. |
| **28** | **Reviewer comment** | 1. Incorporate more infographics and visuals to make the information more accessible and engaging for a wider audience. |
|  | **Author response** | We have now added 2 additional tables and 2 figures to enhance and explain the text:  **Table 2. Information contained within the front pages of the Centre reports** (page 11)  **Table 5. Examples of associated activities conducted by the WCEC** (page 22)  **Fig 2. Questions received, prioritised, and accepted onto the Centre work programme** (page 9)  **Fig 4. Evidence Centre rapid review (evidence synthesis) outputs** (page 20)  The suggestions to use alternative methods of presentation such as videos or are helpful and insightful.  To aid wider accessibility of information related to the Centre processes and outputs, we have added additional information (italics below) and an additional reference (*40), which refers readers to a [public facing legacy report for the Wales COVID-19 Evidence Centre](https://researchwalesevidencecentre.co.uk/sites/default/files/2023-11/Wales_COVID-19_Evidence_Centre_Legacy_Report_June23_ENGLISH.pdf) (WCEC).  ***Wales COVID‐19 Evidence Centre Legacy Report. Wales COVID-19 Evidence Centre. June 2023.** <https://researchwalesevidencecentre.co.uk/sites/default/files/2023-11/Wales_COVID-19_Evidence_Centre_Legacy_Report_June23_ENGLISH.pdf>  (The purpose of this public facing report was to make our work accessible to a wide audience (complies with style guidance for accessible public documents). Here the work of the WCEC, including methodology, reviews, results, knowledge mobilisation and impact are presented in lay language, engaging visuals and infographics)  We have included the following in our Discussion section (page 28, lines 567 to 571):  *To make the work of the WCEC more widely accessible, the Centre also produced a publicly available legacy report.(40) This report includes information about the Centre structure, methods, reviews, knowledge mobilisation and impact. The lay summaries accompanying each review were written by members of the Centre public partnership group, and together with the infographics, increase accessibility of the review findings.* |
| **29** | **Reviewer comment** | 2. Explore different ways to present findings, such as creating poems or videos to cater to various preferences and increase engagement. |
|  | **Author response** | We acknowledge that this is a good idea to increase accessibility and meet the preferences for a wider audience.  Indeed, one of our public group members had suggested poetry for one of our Evidence Centre reports, which focused on children and education during the pandemic. We have now included this in the discussion (page 31 lines 590 - 595):  *A suggestion for wider dissemination of findings in a different format was made by a public member (e.g., review findings relevant to children and young people could be made into a poem). We acknowledge that wider dissemination methods such as creating stories, poems or videos would likely be valuable to make findings more accessible and engaging to wider groups. However, this was not possible at the time due to resource constraints (available staff time and additional funding).*  We have also referenced the knowledge mobilisation model ‘Developing Evidence Enriched Practice’ in the introduction (Page 4, line 95). This includes very engaging methods of sharing research findings and involving people in research and ways to share research findings.  We have already developed videos of our knowledge mobilisation and impact methods and review methods, which are accessible on our new Evidence Centre website. We have included information about this in our discussion (page 31, lines 595-596):  *Videos of our methods relating to knowledge mobilisation, impact and rapid reviews are now available on our new Centre website* |
| **30** | **Reviewer comment** | 3. Enhance Language Accessibility: Simplify language in reports so that key points are clear to all stakeholders, including those with various levels of expertise. |
|  | **Author response** | This is an important point, and the following information is already detailed within our manuscript:  All our reports include a 2-page Topline summary of the key findings at the beginning of each report, which is written in an accessible language. These summaries are reviewed by the stakeholders (not usually scientific or evidence review experts) and members of our public representative group, to ensure that findings are clearly explained.  We have also added the following (page 31, lines 585-589):  *To make the work of the WCEC more widely accessible, the Centre also produced a public facing legacy report.(43) This report includes information about the Centre structure, methods, reviews, knowledge mobilisation and impact in an accessible format. The lay summaries accompanying each review were written by members of the Centre public partnership group, and together with the infographics, increase accessibility of the review findings.*  The WCEC public facing legacy report which we refer readers to, which makes a good companion report to this manuscript and enhances accessibility. This has also been reviewed by our public members for understanding and accessibility. |
| **31** | **Reviewer comment** | 4. Conduct evaluations on different dissemination methods to assess their impact and effectiveness in reaching and engaging stakeholders. |
|  | **Author response** | This is excellent advice.  During the lifetime of the WCEC (2021-2023), we did not conduct evaluations of the different dissemination methods, which we used.  We have made reference to this in the discussion under the limitations section (page 36, line 556 – 558):  *Methods to better evaluate different dissemination methods to assess their impact and effectiveness should have been planned and implemented as part of the processes from the start.* |
| **32** | **Reviewer comment** | 5. Streamline Questions: Formulate more specific and narrow research questions to ensure deliverability and manage expectations throughout the process. |
|  | **Author response** | The research questions, which the Centre received from the stakeholders were typically very broad. The Centre had to work with the stakeholders to manage their expectations, and streamline the questions to ensure that the reviews could deliver useful information and be completed within the stakeholder’s timeframe. The streamlining/focusing of the questions was part of the rapid evidence review process, and not part of the knowledge mobilisation processes of the Centre, which were the focus of this manuscript (and so was not described).  However, we agree that this point would be of interest to readers of this manuscript. Therefore, we have made reference to this in the Discussion section (Page 28, Lines 523 -525), and included a reference to another Centre manuscript, where the streamlining of research questions for the Evidence reviews is described in detail:  *The responsiveness and involvement of stakeholders and members of the public partnership were key to focusing the research questions, which were often too broad to be answered in the available amount of time.(23)*  **A bespoke rapid evidence review process engaging stakeholders for supporting evolving and time-sensitive policy and clinical decision-making: reflection and lessons learned from the Wales Covid-19 Evidence Centre 2021-23. Lewis R et al.** <https://assets-eu.researchsquare.com/files/rs-3878814/v1/59d98322-ac9e-492b-aead-afbd02db0b37.pdf?c=1706010939> |
| **33** | **Reviewer comment** | 6. Involve stakeholders in the knowledge mobilization plan from the beginning to gather valuable insights and ensure relevance. |
|  | **Author response** | This again is excellent point.  In the discussion ‘Strengths and Limitations’ section (page 36. Lines 667 – 669), we have included:  *Lessons that will be taken forward include i) refining our engagement and communication with policy and practice decision makers, ii) involving stakeholders in the development of the knowledge mobilisation plan from the beginning of the collaboration to gather insights and ensure relevance,…’* |
| **34** | **Reviewer comment** | 7. Continue close collaboration with stakeholders, including public partnership members, to ensure that evidence outputs are targeted, timely, and effectively used. |
|  | **Author response** | Thank you for this helpful suggestion. We have now included this point in the manuscript Discussion (page 29, lines 537 - 544):  *Following the end of funding of the Wales COVID-19 Evidence Centre, we received 5 further years of funding and are now funded as the ‘Health and Care Research Wales Evidence Centre (2023-2028).(41) This enables us to continue longer term close collaborations with the stakeholders we have worked with in the COVID-19 Evidence Centre to enhance use of our report findings, and collect and evidence longer term evidence of impact. We also continue to work with public members and currently the new Evidence Centre has a public partnership group of 10 members who are involved in all the Centre processes.* |
| **35** | **Reviewer comment** | 8. Reflect on the impact requirements for academia and explore ways to align rapid reviews with the peer-reviewed journal publication criteria. |
|  | **Author response** | We acknowledge that this is an interesting point and important point. We have now made reference to this in the discussion (Page 35/36, Table 8, last box):  *When producing rapid reviews, the requirements and criteria of peer reviewed journals and the research and impact requirements of academia should be considered. Our review methodology is aligned to recognised rapid review methodology and a number of the reviews have now been published in peer reviewed journals.(23)*  The detailed method for our rapid review process is also described in referenced the manuscript (23):  **A bespoke rapid evidence review process engaging stakeholders for supporting evolving and time-sensitive policy and clinical decision-making: reflection and lessons learned from the Wales Covid-19 Evidence Centre 2021-23. Lewis R et al.** <https://assets-eu.researchsquare.com/files/rs-3878814/v1/59d98322-ac9e-492b-aead-afbd02db0b37.pdf?c=1706010939> |
| **36** | **Reviewer comment** | 9. Develop more robust and long-term engagement strategies with policy and practice decision-makers to enhance the utilization and impact of evidence reviews. |
|  | **Author response** | Thank you for this helpful suggestion. We have included reference to this in the discussion (page 29 lines 537 - 548).  *Following the end of funding of the Wales COVID-19 Evidence Centre, we received 5 further years of funding, and are now funded as the ‘Health and Care Research Wales Evidence Centre (2023-2028).(41) This enables us to continue longer term close collaborations with the stakeholders we have worked with in the COVID-19 Evidence Centre to enhance use of our report findings, and collect and evidence longer term evidence of impact. We also continue to work with public members and currently the new Evidence Centre has a public partnership group of 10 members who are involved in all the Centre processes.*  *To improve engagement and communication with policy and practice decision makers going forward, the new Evidence centre has now implemented ongoing, additional short meetings with stakeholders, which focus on service evaluation, knowledge mobilisation planning and impact tracking. The new Centre also now has additional contact between the review team and stakeholders during the review process (e.g. to discuss the review protocol), and this helps to manage and clarify expectations.* |
| **37** | **Reviewer comment** | 10. Improve communication with stakeholders at all process stages to manage demand effectively and ensure expectation clarity. |
|  | **Author response** | This is a good and astute recommendation from the reviewer.  We have learnt this lesson during the lifetime of the WCEC and noted that improved communication is needed to manage expectations, ensure understanding of the findings and ensure stakeholder support of knowledge mobilisation and impact. We have referenced this in the discussion (page 29, lines 545 - 549).  *To improve engagement and communication with policy and practice decision makers going forward, the new Evidence centre has now implemented ongoing, additional short meetings with stakeholders, which focus on service evaluation, knowledge mobilisation planning and impact tracking. The new Centre also now has additional contact between the review team and stakeholders during the review process (e.g. to discuss the review protocol), and this helps to manage and clarify expectations.* |

# **Appendix 1. Initial Letter to the Author from PLOS ONE**

Thank you for submitting your manuscript to PLOS ONE. After careful consideration, we feel that it has merit but does not fully meet PLOS ONE’s publication criteria as it currently stands. Therefore, we invite you to submit a revised version of the manuscript that addresses the points raised during the review process.

 The insights shared in your manuscript are invaluable, and I appreciate the effort put into documenting this important work.

While the manuscript provides a comprehensive overview of the WCEC's activities, there are some areas where additional detail and clarification would enhance its readability and impact. I have summarized the key points from the reviewers' comments below:

**Enhancement of Methods Section:** Reviewers suggested providing more detail on stakeholder engagement, collaborative work processes, and data collection methods to offer readers a clearer understanding of the WCEC's approach.
Improvement in Presentation: Incorporating more visuals, simplifying language, and exploring alternative presentation methods such as videos or poems could make the information more accessible and engaging for a wider audience.
Streamlining of Content: Streamlining research questions and aligning rapid review processes with publication criteria were recommended to ensure clarity and manage expectations effectively.
Stakeholder Engagement: Continued close collaboration with stakeholders, including policy and practice decision-makers, was emphasized to enhance the utilization and impact of evidence reviews.
I believe that addressing these suggestions will significantly strengthen the manuscript and amplify its contribution to informing health and social care policy and practice during public health emergencies like the COVID-19 pandemic.

Additionally, I would like to propose the inclusion of an article featuring an interview with Prof. Marianna Arvanitakis, the winner of the European Award for the Best Medical Practice in the COVID-19 Pandemic. This article could highlight how recognition of exemplary practices can serve as a valuable resource in crisis situations and inform future health and social care policies. available here: https://globalbioethicsenquiry.com/wp-content/uploads/2023/07/VP2-JASNA.pdf

Thank you for considering these suggestions. I look forward to seeing the revised manuscript and the potential addition of the suggested article.

Best regards,

Journal requirements:

When submitting your revision, we need you to address these additional requirements.

1. Please ensure that your manuscript meets PLOS ONE's style requirements, including those for file naming. The PLOS ONE style templates can be found at
https://journals.plos.org/plosone/s/file?id=wjVg/PLOSOne_formatting_sample_main_body.pdf and
https://journals.plos.org/plosone/s/file?id=ba62/PLOSOne_formatting_sample_title_authors_affiliations.pdf

2. We note that you have indicated that there are restrictions to data sharing for this study. For studies involving human research participant data or other sensitive data, we encourage authors to share de-identified or anonymized data. However, when data cannot be publicly shared for ethical reasons, we allow authors to make their data sets available upon request. For information on unacceptable data access restrictions, please see http://journals.plos.org/plosone/s/data-availability#loc-unacceptable-data-access-restrictions.

Before we proceed with your manuscript, please address the following prompts:

a) If there are ethical or legal restrictions on sharing a de-identified data set, please explain them in detail (e.g., data contain potentially identifying or sensitive patient information, data are owned by a third-party organization, etc.) and who has imposed them (e.g., a Research Ethics Committee or Institutional Review Board, etc.). Please also provide contact information for a data access committee, ethics committee, or other institutional body to which data requests may be sent.

b) If there are no restrictions, please upload the minimal anonymized data set necessary to replicate your study findings to a stable, public repository and provide us with the relevant URLs, DOIs, or accession numbers. Please see http://www.bmj.com/content/340/bmj.c181.long for guidelines on how to de-identify and prepare clinical data for publication. For a list of recommended repositories, please see https://journals.plos.org/plosone/s/recommended-repositories. You also have the option of uploading the data as Supporting Information files, but we would recommend depositing data directly to a data repository if possible.

Please update your Data Availability statement in the submission form accordingly.

[Note: HTML markup is below. Please do not edit.]

Reviewers' comments:

Reviewer's Responses to Questions

**Comments to the Author**

1. Is the manuscript technically sound, and do the data support the conclusions?

The manuscript must describe a technically sound piece of scientific research with data that supports the conclusions. Experiments must have been conducted rigorously, with appropriate controls, replication, and sample sizes. The conclusions must be drawn appropriately based on the data presented.

Reviewer #1: Partly

Reviewer #2: Partly

2. Has the statistical analysis been performed appropriately and rigorously?

Reviewer #1: N/A

Reviewer #2: No

3. Have the authors made all data underlying the findings in their manuscript fully available?

The [PLOS Data policy](https://eur03.safelinks.protection.outlook.com/?url=http%3A%2F%2Fwww.plosone.org%2Fstatic%2Fpolicies.action%23sharing&data=05%7C02%7Ccoopera8%40cardiff.ac.uk%7Cbf3b20094d054de6efdd08dc6de1e483%7Cbdb74b3095684856bdbf06759778fcbc%7C1%7C0%7C638506064322454378%7CUnknown%7CTWFpbGZsb3d8eyJWIjoiMC4wLjAwMDAiLCJQIjoiV2luMzIiLCJBTiI6Ik1haWwiLCJXVCI6Mn0%3D%7C0%7C%7C%7C&sdata=7UWywQDpzpZbPCKMWj7YyG9J4%2BBx2dphMcp6jTm2WsY%3D&reserved=0) requires authors to make all data underlying the findings described in their manuscript fully available without restriction, with rare exception (please refer to the Data Availability Statement in the manuscript PDF file). The data should be provided as part of the manuscript or its supporting information, or deposited to a public repository. For example, in addition to summary statistics, the data points behind means, medians and variance measures should be available. If there are restrictions on publicly sharing data—e.g. participant privacy or use of data from a third party—those must be specified.

Reviewer #1: No

Reviewer #2: No

4. Is the manuscript presented in an intelligible fashion and written in standard English?

PLOS ONE does not copyedit accepted manuscripts, so the language in submitted articles must be clear, correct, and unambiguous. Any typographical or grammatical errors should be corrected at revision, so please note any specific errors here.

Reviewer #1: Yes

Reviewer #2: Yes

5. Review Comments to the Author

Please use the space provided to explain your answers to the questions above. You may also include additional comments for the author, including concerns about dual publication, research ethics, or publication ethics. (Please upload your review as an attachment if it exceeds 20,000 characters)

Reviewer #1: Thank you for the opportunity to review this manuscript, and congratulations to the WCEC for the important work they completed during the COVID-19 pandemic. I appreciate how difficult these types of reports are to write, as they don't fit into the traditional IMRaD format of our typical scientific papers. Overall, I would like to have more detail within the methods section to better understand what steps the WCEC undertook as part of their work and KM. I have provided some specific examples of this below.

Background
- Page 4, Lines 100-102 - The authors make an important point that research evidence is not the only factor that decisions should be made on. I think it would be important to note that values/preferences (of individuals and communities) and also resources (financial, human, etc.) also play an important role in decision-making
- Page 4, Line 103 - this sentence is a bit awkward (acheiving impact is enabled...)

Figure 1: I like the use of the KTA framework, but it would be helpful to have more of the original steps maintained in the figure so the reader can see how the WCEC applied/adapted it for their KM purposes.

Methods
- It would be helpful for the authors to provide more detail on how the various stakeholder groups

In section (Table 1) were engaged. How were questions identified and prioritized?
- I would like to read more about how the WCED and stakeholders worked collaboratively (page 7, Lines 155-162). Were three online meetings completed per review or over the course of the WCEC operations?
- What are some concrete examples of capacity building, sharing methodologies, and identifying gaps as part of the "associated activities" - maybe a table listing these would be helpful?
- Who were the stakeholders who were invited to take part in the survey? Were there any eligibility criteria set out? How were they invited to participate?
- What is meant by "accepted onto the work program" (page 13, line 282-4? Only these questions were taken on by the WCEC or only these questions were evaluated through the survey?
- More detail could be added as to how citations were identified, how email and meeting sessions were used to capture data when surveys were incomplete

Results
- What is meant by "good engagement" - how was this determined vs. poor engagement for example? (page 13, line 294).
- Did all stakeholders take part in all steps (lines 295-299)
- How many reviews were undertaken in total? How many were evaluated through the survey and how many through the other supplementary data collection methods?
- Can you provide demographic data for survey respondents?
- I believe the quotations come from the open ended questions in the survey. How was this qualitative data analysed? (it is not mentioned in the methods)
- SImilar comment on Page 14, Lines 320, what is meant by "worked well" - how was this determined? Two examples are given, but it may be helpful to have this information collated across all reviews and a summary presented

Table 2
- I now see that information on the 51 completed reviews is listed in Table 2 but I think that information should be a key feature in the results
- Timeline of completion - is it possible to give a mean or IQR rather than "usually 1 week to 3 months"
- The authors refer to different parts of the report, (topline summary, etc.). It would be helpful to have described earlier what a typical report included
- This table lists some examples of impact, but it would be more comprehensive if this was collated across reviews, rather than just choosing a few examples (which may be perceived as biasing the anticipated impact)

I'm not clear the distinction between survey results and the "feedback and collection of metrics" section 3.5.2 - how was this data collected if not through the survey?

Table 6 - how was this list compiled? Was a certain process used, or would this be better placed as part of the discussion? In fact, much of it is repeated in the discussion.

Minor suggestions for the authors' consideration
- There are a number of longer sentences, particularly in the background which could be broken up to enhance readability.
- Page 3, Line 88, spell out the acronym for BRIDGE criteria

Reviewer #2: This scoping review focuses on the role of care managers and the knowledge mobilization steps undertaken by the Welsh Centre for Evidence and Dissemination (WCEC) during the COVID-19 pandemic. The WCEC engaged closely with stakeholders, utilized the Knowledge to Action framework, and successfully disseminated evidence reviews to inform health and social care decision-making.
However, some significant concerns need to be addressed before publication.
1. Incorporate more infographics and visuals to make the information more accessible and engaging for a wider audience.
2. Explore different ways to present findings, such as creating poems or videos to cater to various learning preferences and increase engagement.
3. Enhance Language Accessibility: Simplify language in reports so that key points are clear to all stakeholders, including those with various levels of expertise.
4. Conduct evaluations on different dissemination methods to assess their impact and effectiveness in reaching and engaging stakeholders.
5. Streamline Questions: Formulate more specific and narrow research questions to ensure deliverability and manage expectations throughout the process.
6. Involve stakeholders in the knowledge mobilization plan from the beginning to gather valuable insights and ensure relevance.
7. Continue close collaboration with stakeholders, including public partnership members, to ensure that evidence outputs are targeted, timely, and effectively used.
8. Reflect on the impact requirements for academia and explore ways to align rapid reviews with the peer-reviewed journal publication criteria.
9. Develop more robust and long-term engagement strategies with policy and practice decision-makers to enhance the utilization and impact of evidence reviews.
10. Improve communication with stakeholders at all process stages to manage demand effectively and ensure expectation clarity.

6. PLOS authors have the option to publish the peer review history of their article ([what does this mean?](https://eur03.safelinks.protection.outlook.com/?url=https%3A%2F%2Fjournals.plos.org%2Fplosone%2Fs%2Feditorial-and-peer-review-process%23loc-peer-review-history&data=05%7C02%7Ccoopera8%40cardiff.ac.uk%7Cbf3b20094d054de6efdd08dc6de1e483%7Cbdb74b3095684856bdbf06759778fcbc%7C1%7C0%7C638506064322463973%7CUnknown%7CTWFpbGZsb3d8eyJWIjoiMC4wLjAwMDAiLCJQIjoiV2luMzIiLCJBTiI6Ik1haWwiLCJXVCI6Mn0%3D%7C0%7C%7C%7C&sdata=bmMuw2HokgHJjGxn%2FV%2BHKCcQzDpgT%2FBYrXmNzYLNqQw%3D&reserved=0)). If published, this will include your full peer review and any attached files.


If you choose “no”, your identity will remain anonymous but your review may still be made public.

While revising your submission, please upload your figure files to the Preflight Analysis and Conversion Engine (PACE) digital diagnostic tool, [https://pacev2.apexcovantage.com/](https://eur03.safelinks.protection.outlook.com/?url=https%3A%2F%2Fpacev2.apexcovantage.com%2F&data=05%7C02%7Ccoopera8%40cardiff.ac.uk%7Cbf3b20094d054de6efdd08dc6de1e483%7Cbdb74b3095684856bdbf06759778fcbc%7C1%7C0%7C638506064322475905%7CUnknown%7CTWFpbGZsb3d8eyJWIjoiMC4wLjAwMDAiLCJQIjoiV2luMzIiLCJBTiI6Ik1haWwiLCJXVCI6Mn0%3D%7C0%7C%7C%7C&sdata=cnN5cMZo5p4fwBJOLD8oE9P7h%2Bnrywvm12OjQvV50rU%3D&reserved=0). PACE helps ensure that figures meet PLOS requirements. To use PACE, you must first register as a user. Registration is free. Then, login and navigate to the UPLOAD tab, where you will find detailed instructions on how to use the tool. If you encounter any issues or have any questions when using PACE, please email PLOS at [figures@plos.org](mailto:figures@plos.org). Please note that Supporting Information files do not need this step.

# **Appendix 2. Second Letter to the Author from PLOS ONE**

**(Received September 2024)**

PONE-D-23-22096R1

Knowledge mobilisation of rapid evidence reviews to inform health and social care policy and practice in a public health emergency: appraisal of the Wales COVID-19 Evidence Centre processes and impact, 2021-23

PLOS ONE

Dear Dr. Gal,

Thank you for submitting your manuscript to PLOS ONE. After careful consideration, we feel that it has merit but does not fully meet PLOS ONE’s publication criteria as it currently stands. Therefore, we invite you to submit a revised version of the manuscript that addresses the points raised during the review process.

Further to your previous revisions, Reviewer 1 has requested/re-requested some changes to improve the clarity and reporting of your study in this manuscript. Please revise carefully and ensure each comment is addressed in the response to reviewers document submitted with your revised manuscript. 

Please submit your revised manuscript by Oct 25 2024 11:59PM. If you will need more time than this to complete your revisions, please reply to this message or contact the journal office at [plosone@plos.org](mailto:plosone@plos.org). When you're ready to submit your revision, log on to [https://www.editorialmanager.com/pone/](https://eur03.safelinks.protection.outlook.com/?url=https%3A%2F%2Fwww.editorialmanager.com%2Fpone%2F&data=05%7C02%7CGalM%40cardiff.ac.uk%7Cf7ea57011f93454f5fea08dcd275bfbc%7Cbdb74b3095684856bdbf06759778fcbc%7C1%7C0%7C638616650364528694%7CUnknown%7CTWFpbGZsb3d8eyJWIjoiMC4wLjAwMDAiLCJQIjoiV2luMzIiLCJBTiI6Ik1haWwiLCJXVCI6Mn0%3D%7C0%7C%7C%7C&sdata=ROFiKmZNBDWvoXsstWBZ16jVou71XogTaaJyj7zCJEE%3D&reserved=0) and select the 'Submissions Needing Revision' folder to locate your manuscript file.

Please include the following items when submitting your revised manuscript:

- A rebuttal letter that responds to each point raised by the academic editor and reviewer(s). You should upload this letter as a separate file labeled 'Response to Reviewers'.
- A marked-up copy of your manuscript that highlights changes made to the original version. You should upload this as a separate file labeled 'Revised Manuscript with Track Changes'.
- An unmarked version of your revised paper without tracked changes. You should upload this as a separate file labeled 'Manuscript'.

If you would like to make changes to your financial disclosure, please include your updated statement in your cover letter. Guidelines for resubmitting your figure files are available below the reviewer comments at the end of this letter.

If applicable, we recommend that you deposit your laboratory protocols in [protocols.io](https://eur03.safelinks.protection.outlook.com/?url=http%3A%2F%2Fprotocols.io%2F&data=05%7C02%7CGalM%40cardiff.ac.uk%7Cf7ea57011f93454f5fea08dcd275bfbc%7Cbdb74b3095684856bdbf06759778fcbc%7C1%7C0%7C638616650364543357%7CUnknown%7CTWFpbGZsb3d8eyJWIjoiMC4wLjAwMDAiLCJQIjoiV2luMzIiLCJBTiI6Ik1haWwiLCJXVCI6Mn0%3D%7C0%7C%7C%7C&sdata=xGINxGAaaU8Uwtcaxs%2BSqnGYIebWkgxWDvSTy2UOxd8%3D&reserved=0) to enhance the reproducibility of your results. [Protocols.io](https://eur03.safelinks.protection.outlook.com/?url=http%3A%2F%2Fprotocols.io%2F&data=05%7C02%7CGalM%40cardiff.ac.uk%7Cf7ea57011f93454f5fea08dcd275bfbc%7Cbdb74b3095684856bdbf06759778fcbc%7C1%7C0%7C638616650364553614%7CUnknown%7CTWFpbGZsb3d8eyJWIjoiMC4wLjAwMDAiLCJQIjoiV2luMzIiLCJBTiI6Ik1haWwiLCJXVCI6Mn0%3D%7C0%7C%7C%7C&sdata=6X0U%2FF%2FTLTrmcKnFxQIQLF%2F2JY%2F4iH1Awkztp%2FptafI%3D&reserved=0)assigns your protocol its own identifier (DOI) so that it can be cited independently in the future. For instructions see: [https://journals.plos.org/plosone/s/submission-guidelines#loc-laboratory-protocols](https://eur03.safelinks.protection.outlook.com/?url=https%3A%2F%2Fc05y1x9s.r.us-east-2.awstrack.me%2FL0%2Fhttps%3A%252F%252Fjournals.plos.org%252Fplosone%252Fs%252Fsubmission-guidelines%2523loc-laboratory-protocols%2F1%2F010f0191decb0a1c-73e0fe65-1ee0-4635-b293-ed07a91a9309-000000%2FZCc2sxtF3Jm3y6vb42833HVLxKY%3D176&data=05%7C02%7Cgalm%40cardiff.ac.uk%7C072942f1dd7d49020b6208dcd204d3c6%7Cbdb74b3095684856bdbf06759778fcbc%7C1%7C0%7C638616165386232213%7CUnknown%7CTWFpbGZsb3d8eyJWIjoiMC4wLjAwMDAiLCJQIjoiV2luMzIiLCJBTiI6Ik1haWwiLCJXVCI6Mn0%3D%7C0%7C%7C%7C&sdata=FqjRKUyck2Xx5wVoHxqnMcd1S6XaDruDhOxpO5PlmFg%3D&reserved=0). Additionally, PLOS ONE offers an option for publishing peer-reviewed Lab Protocol articles, which describe protocols hosted on [protocols.io](https://eur03.safelinks.protection.outlook.com/?url=http%3A%2F%2Fprotocols.io%2F&data=05%7C02%7CGalM%40cardiff.ac.uk%7Cf7ea57011f93454f5fea08dcd275bfbc%7Cbdb74b3095684856bdbf06759778fcbc%7C1%7C0%7C638616650364562289%7CUnknown%7CTWFpbGZsb3d8eyJWIjoiMC4wLjAwMDAiLCJQIjoiV2luMzIiLCJBTiI6Ik1haWwiLCJXVCI6Mn0%3D%7C0%7C%7C%7C&sdata=EHySL7EGzoYl%2B5nWTLamGEVHuxdi%2BanlNKDdVEyr6Ms%3D&reserved=0). Read more information on sharing protocols at [https://plos.org/protocols?utm_medium=editorial-email&utm_source=authorletters&utm_campaign=protocols](https://eur03.safelinks.protection.outlook.com/?url=https%3A%2F%2Fc05y1x9s.r.us-east-2.awstrack.me%2FL0%2Fhttps%3A%252F%252Fplos.org%252Fprotocols%253Futm_medium%3Deditorial-email%2526utm_source%3Dauthorletters%2526utm_campaign%3Dprotocols%2F1%2F010f0191decb0a1c-73e0fe65-1ee0-4635-b293-ed07a91a9309-000000%2Fy3CLqN5x_gE9eTmco3TgtrLfPSc%3D176&data=05%7C02%7Cgalm%40cardiff.ac.uk%7C072942f1dd7d49020b6208dcd204d3c6%7Cbdb74b3095684856bdbf06759778fcbc%7C1%7C0%7C638616165386246322%7CUnknown%7CTWFpbGZsb3d8eyJWIjoiMC4wLjAwMDAiLCJQIjoiV2luMzIiLCJBTiI6Ik1haWwiLCJXVCI6Mn0%3D%7C0%7C%7C%7C&sdata=Ug8OB4JyRtNMU4Yp%2FQXFBY0hVFCXqk%2BvslKg4sFsh1Q%3D&reserved=0).

We look forward to receiving your revised manuscript.

Kind regards,

Jennifer Tucker, PhD
Staff Editor
PLOS ONE


[Note: HTML markup is below. Please do not edit.]

Reviewers' comments:

Reviewer's Responses to Questions

**Comments to the Author**

1. If the authors have adequately addressed your comments raised in a previous round of review and you feel that this manuscript is now acceptable for publication, you may indicate that here to bypass the “Comments to the Author” section, enter your conflict of interest statement in the “Confidential to Editor” section, and submit your "Accept" recommendation.

Reviewer #1: (No Response)

2. Is the manuscript technically sound, and do the data support the conclusions?

The manuscript must describe a technically sound piece of scientific research with data that supports the conclusions. Experiments must have been conducted rigorously, with appropriate controls, replication, and sample sizes. The conclusions must be drawn appropriately based on the data presented.

Reviewer #1: Partly

3. Has the statistical analysis been performed appropriately and rigorously?

Reviewer #1: N/A

4. Have the authors made all data underlying the findings in their manuscript fully available?

The [PLOS Data policy](https://eur03.safelinks.protection.outlook.com/?url=http%3A%2F%2Fc05y1x9s.r.us-east-2.awstrack.me%2FL0%2Fhttp%3A%252F%252Fwww.plosone.org%252Fstatic%252Fpolicies.action%2523sharing%2F1%2F010f0191decb0a1c-73e0fe65-1ee0-4635-b293-ed07a91a9309-000000%2FgFlHXx4onYJxtxClfbSBLzmtPhU%3D176&data=05%7C02%7Cgalm%40cardiff.ac.uk%7C072942f1dd7d49020b6208dcd204d3c6%7Cbdb74b3095684856bdbf06759778fcbc%7C1%7C0%7C638616165386256045%7CUnknown%7CTWFpbGZsb3d8eyJWIjoiMC4wLjAwMDAiLCJQIjoiV2luMzIiLCJBTiI6Ik1haWwiLCJXVCI6Mn0%3D%7C0%7C%7C%7C&sdata=HwqLr43wsXedXVXbMK3wVelOCxXKfIvhz9XzuzalLrE%3D&reserved=0) requires authors to make all data underlying the findings described in their manuscript fully available without restriction, with rare exception (please refer to the Data Availability Statement in the manuscript PDF file). The data should be provided as part of the manuscript or its supporting information, or deposited to a public repository. For example, in addition to summary statistics, the data points behind means, medians and variance measures should be available. If there are restrictions on publicly sharing data—e.g. participant privacy or use of data from a third party—those must be specified.

Reviewer #1: No

5. Is the manuscript presented in an intelligible fashion and written in standard English?

PLOS ONE does not copyedit accepted manuscripts, so the language in submitted articles must be clear, correct, and unambiguous. Any typographical or grammatical errors should be corrected at revision, so please note any specific errors here.

Reviewer #1: Yes

6. Review Comments to the Author

Please use the space provided to explain your answers to the questions above. You may also include additional comments for the author, including concerns about dual publication, research ethics, or publication ethics. (Please upload your review as an attachment if it exceeds 20,000 characters)

Reviewer #1: Thank you to the authors for their revisisions, the manuscript is much improved.

I have a few additional/outstanding comments.

1. Was ethical approval sought or received for this survey? It is not mentioned anywhere

2. There are some inconsistencies in the use of acronyms throughout (e.g., TAC on line 160 and spelled out on line 165).

3. Line 172 - were questions received and prioritized at one time or on a rolling basis? I am guessing the later but it is not clear.

4. Line 182 - what is the collaborating partner review team?

5. Methods - I'm not sure if collaborating and co-production are the appropriate terms based on what is described (which is understandable given the very tight timelines the team was working on). For example, using the IAP2 framework for engagement, what the authors describe sounds more like "consult" or maybe "involve". Perhaps a definition of co-production and collaboration with citation would be helpful.

6. Section 2.4 - the authors mention gaps were "collated" - how was this done, and what was done with this information? are there any examples you can provide?

7. Line 348 seems to be missing something ("agreeing relevant outcomes")

8. The authors define good vs. poor engagement, but this probably fits better in the methods (if it was prespecified) vs. the results

9. Line 359 - n = 21 responded to the survey, how many were invited?

10. The authors have provided more information as to how the qualitative data were collected, but there is no description of data analysis approaches for the qualitative (or quantitative data for that matter)

11. Line 439 - how many of the 21 surveys were incomplete?

12. Table 7 could be integrated with Table 4, perhaps as a row of "policy impacts"; there is already some duplication in the table (e.g., infographics for midwives).

13. With respect to facilitators to the impact or how to better mobilize next time, where did the resources come from to develop this centre?

14. Table 8 - i'm unclear if these are part of the "results" (under the objective to provide recommendations for best practice" or discussion. Perhaps if the authors were more clear on whether these are personal reflections or more informed by data.

15. The strengths/limitations section should focus more on the strengths/limitations of this paper and its evaluation rather than the centre as a whole.

16. Another pass for readability is advised... for example "it is worth noting" is on line 544 and line 551.

7. PLOS authors have the option to publish the peer review history of their article ([what does this mean?](https://eur03.safelinks.protection.outlook.com/?url=https%3A%2F%2Fc05y1x9s.r.us-east-2.awstrack.me%2FL0%2Fhttps%3A%252F%252Fjournals.plos.org%252Fplosone%252Fs%252Feditorial-and-peer-review-process%2523loc-peer-review-history%2F1%2F010f0191decb0a1c-73e0fe65-1ee0-4635-b293-ed07a91a9309-000000%2Fu2g7eZHWfZ82SL6fjlkJPSsgp8U%3D176&data=05%7C02%7Cgalm%40cardiff.ac.uk%7C072942f1dd7d49020b6208dcd204d3c6%7Cbdb74b3095684856bdbf06759778fcbc%7C1%7C0%7C638616165386263279%7CUnknown%7CTWFpbGZsb3d8eyJWIjoiMC4wLjAwMDAiLCJQIjoiV2luMzIiLCJBTiI6Ik1haWwiLCJXVCI6Mn0%3D%7C0%7C%7C%7C&sdata=1ETiHgzn%2B0kqu0IhZg8pbmn9H1LmkeEJSDQFQS9AQhc%3D&reserved=0)). If published, this will include your full peer review and any attached files.


If you choose “no”, your identity will remain anonymous but your review may still be made public.


**Do you want your identity to be public for this peer review?** For information about this choice, including consent withdrawal, please see our[Privacy Policy](https://eur03.safelinks.protection.outlook.com/?url=https%3A%2F%2Fc05y1x9s.r.us-east-2.awstrack.me%2FL0%2Fhttps%3A%252F%252Fwww.plos.org%252Fprivacy-policy%2F1%2F010f0191decb0a1c-73e0fe65-1ee0-4635-b293-ed07a91a9309-000000%2FmD3rG6jMdnMn7S0IaDiEpMNmt2o%3D176&data=05%7C02%7Cgalm%40cardiff.ac.uk%7C072942f1dd7d49020b6208dcd204d3c6%7Cbdb74b3095684856bdbf06759778fcbc%7C1%7C0%7C638616165386269960%7CUnknown%7CTWFpbGZsb3d8eyJWIjoiMC4wLjAwMDAiLCJQIjoiV2luMzIiLCJBTiI6Ik1haWwiLCJXVCI6Mn0%3D%7C0%7C%7C%7C&sdata=frAZiQFWtaD7ri6g2v5YejuOJD9kuwi%2BvRPEHCj1omY%3D&reserved=0).

Reviewer #1: No

[NOTE: If reviewer comments were submitted as an attachment file, they will be attached to this email and accessible via the submission site. Please log into your account, locate the manuscript record, and check for the action link "View Attachments". If this link does not appear, there are no attachment files.]

While revising your submission, please upload your figure files to the Preflight Analysis and Conversion Engine (PACE) digital diagnostic tool, [https://pacev2.apexcovantage.com/](https://eur03.safelinks.protection.outlook.com/?url=https%3A%2F%2Fc05y1x9s.r.us-east-2.awstrack.me%2FL0%2Fhttps%3A%252F%252Fpacev2.apexcovantage.com%252F%2F1%2F010f0191decb0a1c-73e0fe65-1ee0-4635-b293-ed07a91a9309-000000%2FUwcuhexVmVe2AiSIe7SlMagUZ6E%3D176&data=05%7C02%7Cgalm%40cardiff.ac.uk%7C072942f1dd7d49020b6208dcd204d3c6%7Cbdb74b3095684856bdbf06759778fcbc%7C1%7C0%7C638616165386276117%7CUnknown%7CTWFpbGZsb3d8eyJWIjoiMC4wLjAwMDAiLCJQIjoiV2luMzIiLCJBTiI6Ik1haWwiLCJXVCI6Mn0%3D%7C0%7C%7C%7C&sdata=lXe7WRmcAYcDkk34ls1LhxHEx3f84LQ8YgfmFz1libc%3D&reserved=0). PACE helps ensure that figures meet PLOS requirements. To use PACE, you must first register as a user. Registration is free. Then, login and navigate to the UPLOAD tab, where you will find detailed instructions on how to use the tool. If you encounter any issues or have any questions when using PACE, please email PLOS at [figures@plos.org](mailto:figures@plos.org). Please note that Supporting Information files do not need this step.
